# Circadian Disruption as a Determinant of the Tumor Temporal State in Colorectal Cancer: A PRISMA-Based Systematic Review Integrating Metabolism, Immunity, and Metastasis

**DOI:** 10.3390/ijms27146164

**Published:** 2026-07-10

**Authors:** Mirosław Tarasewicz, Edyta Zbroch, Adam R. Markowski

**Affiliations:** Department of Hypertensiology, Gastroenterology and Internal Medicine, Medical University of Bialystok, 14 Żurawia Street, 15-540 Bialystok, Poland; miroslaw.tarasewicz@umb.edu.pl (M.T.); edyta.zbroch@umb.edu.pl (E.Z.)

**Keywords:** circadian disruption, colorectal cancer, chronotherapy, tumor microenvironment, metastasis, circadian rhythm, tumor temporal state

## Abstract

Circadian rhythms synchronize physiological processes with the light–dark cycle and regulate biological functions relevant to cancer, including cell-cycle control, metabolism, DNA repair, immunity, and tissue homeostasis. Growing evidence indicates that disruption of these temporal mechanisms contributes to tumor initiation, progression, metastasis, and treatment response. In colorectal cancer (CRC), circadian clock dysregulation has emerged as an important component of tumor biology. A systematic search identified 1338 records, of which 43 studies met the eligibility criteria (20 human, 19 experimental, and 4 chronotherapy studies). Across the included studies, statistically significant associations were consistently reported between dysregulation of clock genes such as *PER1*, *PER3*, *CLOCK*, *BMAL1*, *CRY1*, *TIMELESS*, and *ARNTL2* and alterations in proliferation, metabolism, epithelial plasticity, immune regulation, metastatic potential, and treatment responsiveness. Experimental evidence also supported interactions with Wnt signaling, ferroptosis, oxidative-stress adaptation, epithelial–mesenchymal remodeling, and a proposed clock–microbiota–immune axis. Overall, the available evidence indicates that circadian dysregulation represents a systems-level disturbance that gives rise to a multidimensional biological condition, here referred to as the Tumor Temporal State, integrating the metabolic, immune, invasive, and therapeutic dimensions of colorectal cancer biology.

## 1. Introduction

Circadian rhythms are endogenous oscillations with an approximately 24-h periodicity that coordinate physiological processes across tissues and organ systems, enabling organisms to anticipate and adapt to predictable environmental changes [1,2]. In mammals, circadian timing is organized hierarchically by the suprachiasmatic nucleus, which synchronizes peripheral clocks through neural, endocrine, metabolic, and behavioral signals. At the cellular level, rhythmic regulation is generated by interconnected transcription–translation feedback loops centered on the CLOCK–BMAL1 complex and its downstream targets, including the period (*PER1–3*) and cryptochrome (*CRY1–2*) gene families, with additional modulation provided by REV-ERB, ROR, and post-translational regulatory mechanisms [1,3].

Although circadian regulation was initially recognized for its role in sleep–wake physiology, it is now understood to influence numerous biological processes directly involved in carcinogenesis, including cell-cycle control, DNA repair, oxidative-stress responses, mitochondrial metabolism, immune-cell trafficking, epithelial regeneration, and stem-cell maintenance [2,4]. Consequently, disruption of temporal organization may simultaneously influence multiple hallmarks of cancer. Across a broad spectrum of malignancies, altered expression of core clock genes and impaired rhythmic regulation have been associated with tumor initiation, progression, treatment resistance, and metastatic dissemination [4,5]. Taken together, these observations indicate that circadian dysregulation is better viewed as a systems-level disturbance than as an isolated molecular abnormality, capable of reshaping proliferative, metabolic, immune, and adaptive tumor programs [4].

Colorectal cancer (CRC) represents a particularly informative model for investigating these mechanisms because the intestinal epithelium exhibits rapid cellular turnover, intense metabolic activity, continuous interactions with the gut microbiota, and feeding–fasting regulation that is tightly coupled to circadian timing. Experimental studies have demonstrated that disruption of intestinal clock function impairs epithelial homeostasis, compromises barrier integrity, alters inflammatory signaling, and disrupts intestinal stem-cell function, thereby promoting conditions favorable for tumor initiation and progression [6,7,8].

Clinical studies have consistently demonstrated reduced expression of *PER1* and *PER3* in colorectal tumors, supporting disruption of the molecular circadian clock during CRC development [9,10,11]. Increased expression of *CLOCK*, together with altered regulation of additional circadian genes, including *CRY1* and *CRY2*, has also been reported and associated with tumor progression, prognosis, and anatomical tumor location [12,13,14,15,16].

More recently, attention has shifted toward microbiota–immune–metabolic interactions through which circadian regulation may influence tumor behavior [17]. Experimental studies have further demonstrated that circadian dysregulation actively contributes to colorectal cancer progression by promoting metabolic rewiring, proliferation, invasion, and metastatic competence through interactions with oncogenic signaling pathways and the tumor microenvironment [18,19,20,21]. These observations have stimulated growing interest in chronotherapy, circadian biomarkers, and rhythm-restoring therapeutic strategies as emerging components of precision oncology [22,23,24].

Despite substantial progress in recent years, knowledge remains dispersed across experimental, translational, computational, and clinical studies. Consequently, circadian regulation is often discussed within individual biological domains—including metabolism, immunity, metastasis, or treatment response—rather than as an integrated determinant of colorectal cancer biology.

To our knowledge, this is the first PRISMA-based systematic review integrating experimental, translational, and clinical evidence on circadian regulation in colorectal cancer. Rather than proposing a validated biological classification, this review introduces a hypothesis-generating conceptual framework in which circadian dysregulation is viewed as a systems-level disturbance giving rise to a multidimensional biological condition, referred to throughout this review as the Tumor Temporal State. This concept integrates coordinated alterations in metabolism, immune regulation, epithelial plasticity, metastatic competence, and therapeutic responsiveness into a unified systems-level model of colorectal cancer. The proposed Tumor Temporal State should therefore be interpreted as a conceptual model rather than a molecular subtype, biomarker, or clinical classification system.

## 2. Methods

### 2.1. Search Strategy

This systematic review was conducted in accordance with the Preferred Reporting Items for Systematic Reviews and Meta-Analyses (PRISMA 2020) statement. The review was not prospectively registered, and no formal review protocol was prepared because the study was designed as a qualitative synthesis of heterogeneous experimental, translational, and clinical evidence rather than a quantitative evidence synthesis.

**Figure 1:**
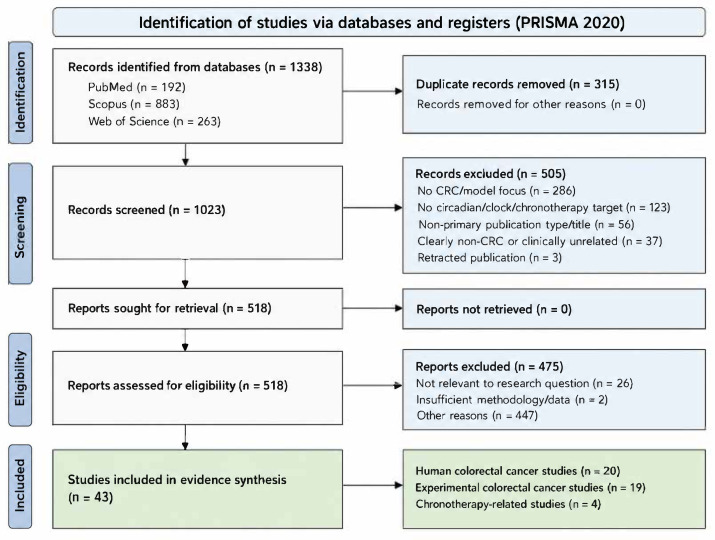
PRISMA 2020 flow diagram of study selection. A total of 1338 records were identified through searches of PubMed/MEDLINE, Scopus, and Web of Science. After removal of 315 duplicate records, 1023 unique records underwent title and abstract screening. At the screening stage, 505 records were excluded and 518 reports were retained for full-text eligibility assessment. Following eligibility review, 475 reports were excluded, resulting in 43 studies included in the final evidence synthesis. Included studies were categorized as human colorectal cancer studies (*n* = 20), experimental colorectal cancer studies (*n* = 19), and chronotherapy-related investigations (*n* = 4). Study selection was performed according to the PRISMA 2020 guidelines.

A comprehensive literature search was conducted in PubMed/MEDLINE, Scopus, and Web of Science from database inception through May 2026. The search strategy combined controlled vocabulary terms with free-text keywords to identify studies examining circadian regulation in colorectal cancer, including terms related to colorectal cancer, circadian rhythms, circadian clock genes, chronobiology, and chronotherapy. Search terms included combinations of “colorectal cancer”, “colon cancer”, “rectal cancer”, “circadian rhythm”, “circadian clock”, “chronotherapy”, “*CLOCK*”, “*BMAL1*”, “*ARNTL*”, “*ARNTL2*”, “*CRY1*”, “*CRY2*”, “*PER1*”, “*PER2*”, “*PER3*”, and “*TIMELESS*”. The complete database-specific search strategies for all electronic databases are provided in Supplementary Table S3. Boolean operators and database-specific adaptations were applied where appropriate. Reference lists of eligible articles were also screened to identify relevant studies not captured through the electronic search. Only peer-reviewed articles published in English were considered. The study selection process is summarized in the PRISMA 2020 flow diagram (Figure 1). The completed PRISMA 2020 checklist is provided as Supplementary Table S5.

To maximize transparency and reproducibility, all retrieved records were independently screened according to predefined eligibility criteria, and disagreements were resolved by consensus after full-text evaluation.

### 2.2. Eligibility Criteria

Studies were eligible for inclusion if they examined circadian regulation, clock-gene dysregulation, chronobiological mechanisms, or chronotherapy-related phenomena in colorectal cancer and reported original experimental, translational, or clinical data. Eligible investigations included studies conducted in human colorectal cancer tissues, clinical cohorts, colorectal cancer cell lines, organoid systems, animal models, xenografts, or chronotherapy-oriented clinical settings. Eligible studies were required to report at least one biological, pathological, metabolic, immune-related, prognostic, metastatic, or therapeutic outcome relevant to colorectal cancer.

Review articles, systematic reviews, meta-analyses, editorials, commentaries, conference abstracts without full-text publication, bibliometric studies, retracted publications, and reports lacking sufficient methodological detail or outcome data were excluded. Studies focusing exclusively on non-colorectal malignancies were also excluded unless colorectal cancer-specific findings were reported separately.

**Figure 2:**
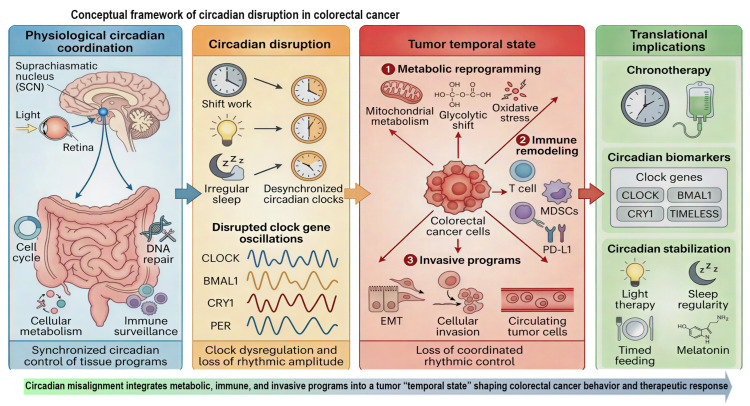
Conceptual framework of circadian disruption in colorectal cancer. Circadian timing coordinates epithelial, metabolic, and immune processes through rhythmic oscillations. Disruption of this temporal coordination gives rise to the Tumor Temporal State, defined here as the overall biological consequence of impaired temporal organization across tumor and host systems. This state is associated with coordinated metabolic, immune, and invasive programs and should be interpreted as a conceptual continuum ranging from preserved rhythmic organization to profound circadian disruption rather than as a binary classification. The proposed Tumor Temporal State provides a conceptual model linking circadian biology with biomarker development, chronotherapy, and rhythm-restoring therapeutic strategies as emerging components of precision oncology. The model is intended as a hypothesis-generating synthesis of the evidence reviewed in this study rather than as a validated biological classification or clinically applicable stratification system. MDSCs—myeloid-derived suppressor cells. PD-L1—programmed death-ligand 1. EMT—epithelial–mesenchymal transition.

Throughout the selection process, particular attention was paid to avoiding duplicate reporting of the same experimental cohorts or overlapping datasets. The conceptual framework integrating circadian disruption with the proposed Tumor Temporal State is presented in Figure 2.

### 2.3. Study Selection and Data Extraction

The database search identified 1338 records across PubMed/MEDLINE, Scopus, and Web of Science. After removal of 315 duplicate records, 1023 unique studies underwent title and abstract screening. This initial screening excluded 505 records that were not relevant to the review question. The remaining 518 articles were assessed in full text. Following detailed evaluation, 475 studies were excluded because they did not meet the eligibility criteria or lacked sufficient methodological rigor. Ultimately, 43 studies were included in the final evidence synthesis. The study selection process is presented in Figure 1.

Records retrieved from all database searches were merged into a single screening dataset and deduplicated before eligibility assessment. Two reviewers independently screened titles, abstracts, and full-text articles according to the predefined eligibility criteria. Any disagreements were resolved through discussion and consensus. Data extraction was conducted independently by the same reviewers using a structured extraction framework. Variables for data extraction were predefined before study selection to minimize selection bias and ensure consistency between reviewers.

Extracted data included publication year, study design, biological model, circadian target, principal findings, and translational relevance. Additional information regarding metastatic behavior, metabolic regulation, immune interactions, prognostic significance, and treatment-related outcomes was collected whenever available. Detailed characteristics of all included studies are provided in Supplementary Table S1.

To facilitate interpretation of the heterogeneous literature, studies were grouped into three predefined evidence domains: (1) human colorectal cancer studies, (2) experimental colorectal cancer studies, and (3) chronotherapy-related investigations. This approach enabled separation of clinical observations, mechanistic experimental findings, and treatment-timing studies while preserving integration across biological levels. Of the 43 included studies, 20 were classified as human colorectal cancer investigations, 19 as experimental studies, and 4 as chronotherapy-related studies.

### 2.4. Risk of Bias and Evidence Classification

The included literature comprised laboratory experiments, translational investigations, animal studies, computational analyses, and observational clinical cohorts. Given this methodological diversity, the use of a single standardized risk-of-bias instrument across all study designs was not considered appropriate. Instead, studies were appraised according to study-specific methodological characteristics and their overall contribution to the evidence base.

Studies were first classified into three predefined evidence domains: human colorectal cancer studies, experimental colorectal cancer studies, and chronotherapy-related investigations. Within each domain, evidence was interpreted according to study design, biological validation, mechanistic depth, reproducibility, and translational relevance.

For observational clinical studies, methodological quality was evaluated using criteria adapted from the Newcastle–Ottawa Scale, including study selection, comparability of cohorts, outcome assessment, adequacy of follow-up, and overall methodological quality. Experimental cell-culture studies were qualitatively assessed with respect to experimental design, biological validation, mechanistic depth, and reproducibility. Animal studies were additionally evaluated according to model relevance and translational applicability to human colorectal cancer. Chronotherapy studies were assessed with particular attention to study design, timing of therapeutic interventions, clinical outcome measures, and applicability to clinical practice.

Risk-of-bias assessment and evidence appraisal were performed independently by two reviewers, with disagreements resolved through discussion and consensus. The results of methodological appraisal and evidence classification are summarized in Supplementary Table S2.

Because the included studies differed substantially in study design, biological models, investigated circadian components, outcome measures, and statistical reporting, quantitative meta-analysis and formal comparison of effect sizes were not considered methodologically appropriate. Likewise, formal quantitative assessment of publication bias or certainty of evidence (e.g., funnel plots or GRADE certainty assessment) was not performed, as these approaches are not suitable for highly heterogeneous bodies of evidence. Consequently, findings were synthesized qualitatively, with particular emphasis on statistically significant associations reported by the original studies whenever available.

The methodological approach adopted in this review was intended to facilitate biologically informed interpretation of heterogeneous evidence rather than a quantitative comparison across fundamentally different study designs. Accordingly, the quality assessment was intended to support interpretation of the evidence rather than generate quantitative quality scores.

## 3. Results

### 3.1. The Structure of the Circadian Clock

Circadian regulation provides the temporal framework that enables organisms to anticipate predictable environmental changes and coordinate physiological processes accordingly. In mammals, this system is organized hierarchically, with the suprachiasmatic nucleus synchronizing peripheral clocks distributed throughout tissues and organs. Although light is the principal entrainment cue, feeding behavior, metabolic signals, hormonal fluctuations, and microbiota-derived metabolites also contribute to the synchronization of circadian activity across the organism [1,2].

At the cellular level, circadian rhythms are generated by interconnected transcription–translation feedback loops centered on the CLOCK–BMAL1 complex. Activation of the period (*PER1–3*) and cryptochrome (*CRY1–2*) genes leads to accumulation of their protein products, which subsequently inhibit CLOCK–BMAL1 activity, thereby generating self-sustained oscillations. Additional regulatory pathways involving the REV-ERB and ROR families of transcription factors further modulate rhythm stability, oscillation amplitude, and phase coordination [2,3].

The biological significance of circadian regulation extends well beyond the maintenance of temporal homeostasis. Core clock pathways regulate numerous processes directly involved in carcinogenesis, including cell-cycle progression, DNA repair, mitochondrial function, oxidative-stress responses, epithelial regeneration, and immune activity [2]. Consequently, disruption of circadian organization has the potential to affect multiple biological processes simultaneously rather than produce isolated molecular abnormalities.

This systems-level organization is particularly relevant in colorectal cancer. The intestinal epithelium undergoes rapid cellular turnover, maintains continuous interactions with the gut microbiota, and is strongly influenced by metabolic and feeding-related signals, all of which are tightly coupled to circadian regulation [6,7,8]. Experimental studies have demonstrated that disruption of intestinal clock function compromises epithelial homeostasis, impairs barrier integrity, alters inflammatory signaling, and affects intestinal stem-cell behavior, thereby creating conditions that may promote tumor initiation and progression [6,7,8]. Beyond these local effects, disruption of rhythmic coordination may also influence key tumor-associated processes, including metabolic adaptation, immune remodeling, invasive behavior, and therapeutic responsiveness [18,19,20,21,25].

Taken together, current evidence indicates that circadian dysregulation in colorectal cancer is best understood as a disturbance of temporal organization operating across multiple biological levels rather than as an isolated defect of individual clock genes. Within the conceptual framework proposed in this review, this systems-level disturbance gives rise to the Tumor Temporal State, which provides the biological framework for the following sections examining its relationships with tumor progression, metastatic competence, immune remodeling, metabolic adaptation, and therapeutic responsiveness.

### 3.2. The Role of Circadian Rhythms in Colorectal Cancer

Clinical and functional studies consistently indicate that circadian dysregulation is a recurrent feature of colorectal cancer and influences multiple dimensions of tumor biology. Although individual investigations have focused on different molecular clock components and diverse biological endpoints, several common themes emerge across the available literature, including disruption of core clock-gene expression, altered regulation of tumor growth and metabolism, and integration of circadian pathways into broader oncogenic networks.

One of the most consistent observations in human CRC is deregulation of key components of the molecular clock. Reduced expression of *PER1* and *PER3* has been consistently reported in colorectal tumors [9,10,11]. Altered expression of *CLOCK*, *CRY1*, *CRY2*, and *ARNTL2* further indicates that circadian dysregulation affects multiple elements of the molecular clock rather than isolated genes [12,13,14,26]. In addition to transcriptional alterations, somatic *CLOCK* mutations have been identified in CRC, suggesting that circadian regulators themselves may become direct targets of tumor evolution [27].

These molecular alterations are accompanied by clinically meaningful associations. Reduced *PER1* and *PER3* expression, increased *CRY1* activity, and broader clock-gene deregulation have been linked to tumor progression, adverse clinicopathological characteristics, and unfavorable clinical outcomes [9,11,15,28]. Several studies further support the prognostic value of circadian pathways, demonstrating associations between clock-gene expression profiles and patient survival or disease behavior. Interestingly, sex-specific relationships between circadian markers and clinical outcomes have also been described, suggesting that host-related biological factors may influence the consequences of clock dysfunction in CRC [28,29].

Mechanistic studies further support the concept that circadian disruption plays an active role in colorectal carcinogenesis. In the *Apc*^Min/+^ mouse model, mutation of the core clock component *PER2* accelerated intestinal tumor development, indicating that circadian regulators may contribute directly to tumor initiation rather than simply reflect established disease [30].

**Figure 3:**
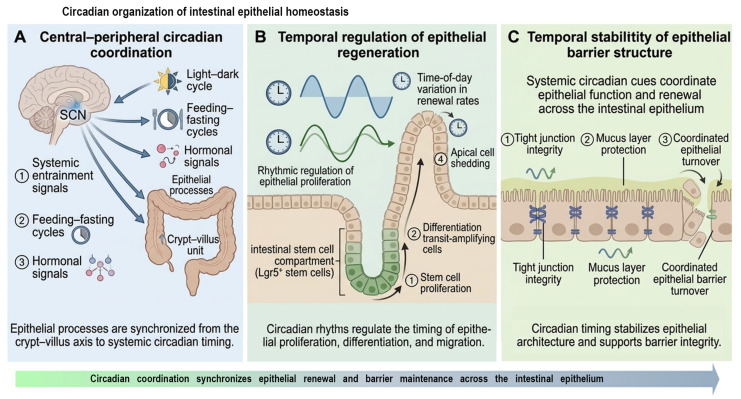
Circadian organization of intestinal epithelial homeostasis. (**A**) Central–peripheral circadian coordination aligns epithelial processes along the crypt–villus axis. (**B**) Circadian regulation controls epithelial renewal, including proliferation, differentiation, migration, and shedding. (**C**) Circadian cues maintain barrier integrity through coordinated regulation of tight junctions, mucus protection, and epithelial turnover.

Beyond changes in clock-gene expression, circadian regulation plays an important role in controlling tumor growth, metabolism, and cellular adaptation. Experimental studies consistently support a tumor-suppressive function of BMAL1-dependent signaling. Loss of BMAL1 activity promotes proliferative and metabolic reprogramming, enhances adaptation to cellular stress, alters Wnt-dependent pathways involved in epithelial homeostasis and stem-cell regulation, and disrupts the balance between the AKT/mTOR and P53/P21 signaling pathways that govern cell-cycle progression and cell-fate decisions [6,18,31,32]. Additional investigations have demonstrated interactions between circadian regulators, microRNA networks, and transcriptional programs controlling differentiation, stress adaptation, and metabolic plasticity, indicating that the biological consequences of circadian disruption extend well beyond the canonical CLOCK–BMAL1 feedback loop [33,34,35]. The principal mechanisms linking circadian regulation with epithelial homeostasis and proliferative control in CRC are summarized in Figure 3.

The available evidence also highlights extensive crosstalk between circadian pathways and established oncogenic programs. TIMELESS promotes tumor growth and progression through epigenetic remodeling and interactions with cytoskeletal regulatory networks [19], whereas reciprocal interactions between clock genes and metastasis-associated regulators such as *MACC1* indicate that circadian dysfunction becomes integrated into broader transcriptional programs governing tumor adaptation and dissemination [20]. Similarly, the BMAL1–REV-ERBα axis has been implicated in angiogenic signaling, metabolic adaptation, and tumor–microenvironment interactions [36,37].

Clinical observations indicate that the influence of circadian dysregulation extends beyond tumor biology itself and may affect therapeutic responsiveness. Altered clock-gene expression has been associated with differential responses to chemotherapy and radiochemotherapy, whereas genetic variation within circadian pathways may contribute to interindividual differences in treatment efficacy, treatment-related toxicity, and long-term clinical outcomes [38,39,40]. Furthermore, analyses of metastatic CRC cohorts demonstrated that circadian-gene deregulation persists throughout disease progression, suggesting that disruption of rhythmic regulation remains biologically relevant even in advanced-stage tumors [41].

Recent transcriptomic and computational studies further support the concept that circadian regulation represents an organizational layer of tumor biology rather than a collection of independent molecular pathways. Integrated circadian-expression signatures have been associated with metabolic remodeling, immune regulation, and aggressive tumor phenotypes, supporting the view that loss of temporal coordination constitutes an additional dimension of colorectal cancer heterogeneity [42,43].

Collectively, these findings indicate that circadian dysregulation in CRC involves coordinated alterations in clock-gene activity, metabolism, oncogenic signaling, and therapeutic responsiveness. Rather than acting through a single molecular pathway, circadian regulation influences multiple interconnected biological processes that collectively shape tumor behavior. These observations further support the concept that circadian dysregulation represents an integral component of colorectal cancer biology rather than merely a secondary consequence of tumor progression.

### 3.3. Circadian Control of Metastatic Competence

Building upon these observations, accumulating evidence indicates that circadian dysregulation contributes to metastatic competence through coordinated mechanisms operating across multiple biological levels. Instead of influencing a single step of the metastatic cascade, disruption of temporal regulation affects tumor-cell plasticity, host–microbiota interactions, and immune surveillance, collectively creating conditions that favor tumor dissemination and metastatic colonization.

Across both clinical and experimental studies, dysfunction of the molecular clock has consistently been associated with invasive tumor phenotypes. Altered expression of *CLOCK* and *CRY1* has been linked to adverse clinicopathological characteristics, lymph-node involvement, and increased migratory potential, suggesting that dysregulation of core clock components accompanies disease progression [15,44]. Similar findings have been reported in metastatic CRC cohorts, where circadian-gene deregulation remains evident in advanced-stage disease, indicating that disruption of temporal regulation persists throughout tumor evolution [41].

Mechanistic investigations provide additional support for these clinical observations. TIMELESS has emerged as a key mediator of invasion-related phenotypes, with experimental studies demonstrating that its upregulation enhances migration and invasion through epigenetic remodeling and MYH9-associated signaling pathways [19]. Circadian regulators also interact with established metastasis-associated genes such as *MACC1*, indicating that clock dysfunction becomes integrated into broader transcriptional programs governing tumor invasion and dissemination [20]. Furthermore, circadian regulation has been linked to stemness-associated traits, cellular plasticity, and adaptation to environmental stress, all of which may facilitate survival during metastatic progression [45,46,47].

Importantly, metastatic competence is shaped not only by tumor-intrinsic mechanisms but also by circadian regulation of host physiology. Several studies have demonstrated that disruption of circadian rhythms alters intestinal barrier integrity, microbial composition, and microbiota-derived signaling pathways [7,8]. These observations suggest that clock dysfunction remodels the biological environment in which tumors develop, extending its influence well beyond malignant cells themselves.

**Figure 4:**
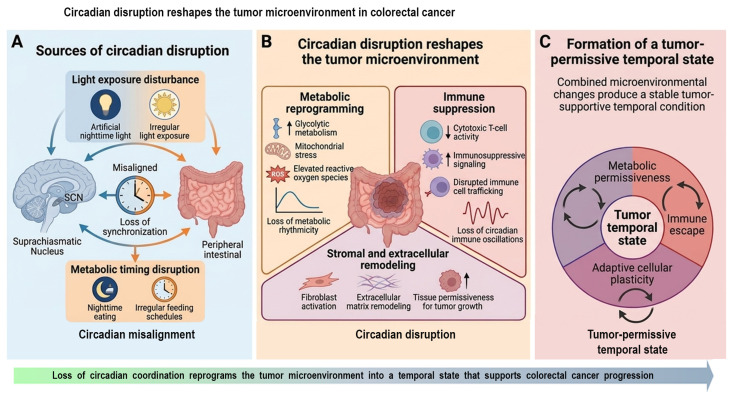
Circadian disruption reshapes the tumor microenvironment in colorectal cancer. (**A**) Environmental and behavioral factors disrupt synchronization between central and peripheral circadian clocks. (**B**) Circadian disruption is associated with coordinated metabolic, immune, and stromal alterations within the tumor microenvironment. (**C**) These changes define a tumor-supportive state characterized by metabolic adaptation, immune modulation, and cellular plasticity.

Particularly compelling is the emerging role of the clock–microbiota–immune axis. Experimental evidence indicates that altered circadian regulation influences microbial metabolite production and bile-acid signaling, leading to the accumulation and metabolic reprogramming of myeloid-derived suppressor cells, thereby contributing to the establishment of permissive pre-metastatic and metastatic niches. Together with studies linking circadian disruption to altered immune-cell trafficking and inflammatory signaling [21,25], these findings support a model in which loss of temporal coordination promotes metastatic progression through coordinated immune remodeling. The effects of circadian disruption on immune and stromal components of the tumor microenvironment are illustrated in Figure 4.

**Figure 5:**
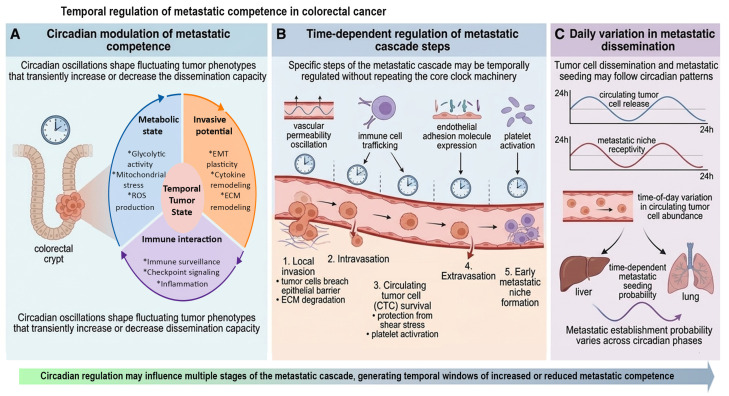
Temporal regulation of metastatic competence in colorectal cancer. (**A**) Circadian rhythms generate dynamic tumor states associated with fluctuating invasive potential. (**B**) Key steps of the metastatic cascade may be temporally regulated. (**C**) Circadian variation in tumor cell dissemination and niche receptivity defines time-dependent windows of metastatic seeding. ECM, extracellular matrix.

Recent investigations further indicate that circadian pathways contribute to metastatic fitness by regulating cellular responses to metabolic and therapeutic stress. Circadian control of oxidative-stress adaptation, ferroptosis susceptibility, and metabolic flexibility may increase the capacity of disseminated tumor cells to survive the unfavorable microenvironment encountered during metastatic spread [48,49,50]. These observations extend the role of circadian regulation beyond tumor invasion alone and implicate temporal organization in multiple stages of the metastatic process. The proposed relationship between circadian dysregulation and metastatic competence is summarized in Figure 5.

Taken together, current evidence supports a model in which metastatic competence emerges through coordinated interactions among tumor-cell plasticity, microbiota-dependent signaling, and immune regulation.

### 3.4. Chronotherapy and Clinical Relevance of Circadian Dysregulation

Beyond its role in tumor progression and metastatic dissemination, circadian regulation also has important implications for therapeutic responsiveness in colorectal cancer. Rather than acting through a single biological pathway, circadian mechanisms intersect with cell-cycle regulation, DNA-damage responses, oxidative-stress adaptation, metabolism, and tumor–microenvironment interactions. Consequently, disruption of temporal organization may affect both intrinsic treatment sensitivity and the capacity of tumor cells to adapt to therapeutic stress.

One of the most consistent findings from experimental studies is the central role of BMAL1-dependent signaling in maintaining treatment responsiveness. Alterations in BMAL1 activity influence cell-cycle progression, apoptosis, DNA-damage responses, and metabolic adaptation, thereby modifying susceptibility to anticancer therapies [31,51]. Consistent with these observations, disruption of rhythmic regulation promotes adaptive responses that favor tumor-cell survival, whereas preservation of circadian integrity is associated with greater therapeutic vulnerability. Experimental evidence has further demonstrated that circadian control contributes to regulation of the metabolic phenotype through HKDC1-dependent pathways, thereby influencing both tumor progression and responsiveness to anticancer treatment [18].

The influence of circadian regulation extends beyond malignant cells to stromal and microenvironmental compartments. Interactions between colorectal cancer cells and tumor-associated fibroblasts have been shown to disrupt circadian organization, alter metabolic activity, reduce apoptosis, and increase resistance to cytotoxic therapy, thereby promoting more aggressive tumor phenotypes [37]. These findings indicate that disruption of temporal coordination may influence treatment responsiveness not only through tumor-intrinsic mechanisms but also through bidirectional communication between malignant cells and their surrounding microenvironment.

Several studies have further implicated circadian pathways in treatment resistance through mechanisms involving metabolic adaptation and cellular stress responses. *ARNTL2* has emerged as a particularly important regulator of oxidative-stress responses and ferroptosis susceptibility. Increased *ARNTL2* activity promotes redox adaptation and cellular survival through modulation of SLC7A11-dependent pathways, thereby reducing sensitivity to therapeutic stress [50]. Likewise, restoration of *NDRG2*-associated circadian signaling has been shown to enhance oxaliplatin responsiveness, highlighting the therapeutic value of preserving circadian integrity [49]. Collectively, these findings indicate that circadian pathways influence not only how tumors respond to treatment but also how effectively they adapt to treatment-induced stress.

Clinical and translational investigations further support the biological relevance of these mechanisms. Associations have been reported between clock-gene expression patterns and responses to chemotherapy or radiochemotherapy, while genetic variation within circadian pathways may contribute to interindividual differences in treatment efficacy and treatment-related toxicity [38,39,40]. These observations indicate that circadian regulation represents an important, yet frequently underrecognized, source of biological variability among patients with colorectal cancer.

Recognition that treatment timing may influence therapeutic outcomes has provided the biological foundation for chronotherapy. Early clinical and translational studies demonstrated that administration of anticancer treatment according to predefined circadian schedules can improve treatment tolerability while maintaining therapeutic efficacy [52,53]. More recent investigations suggest that individual circadian characteristics influence the magnitude of chronotherapeutic benefit and may partly explain variability in treatment-related toxicity and clinical outcomes [54,55]. Together, these findings support the concept that treatment timing should be regarded as a biologically meaningful therapeutic variable rather than merely a logistical consideration.

**Figure 6:**
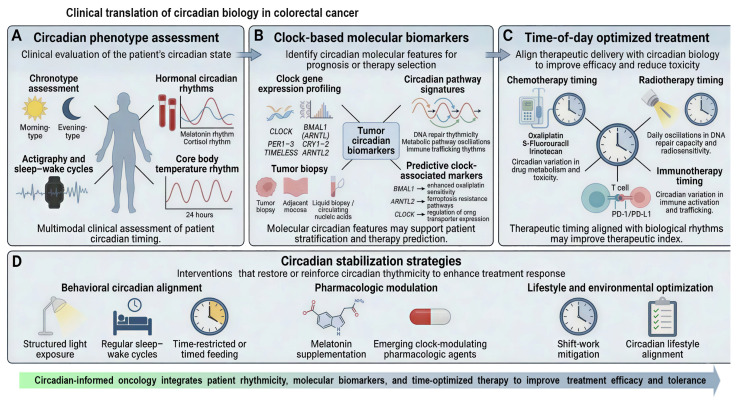
Clinical translation of circadian biology in colorectal cancer. (**A**) Circadian phenotype assessment integrates clinical, behavioral, and systemic markers of rhythmicity. (**B**) Clock-based molecular biomarkers reflect circadian regulation in tumor and host tissues. (**C**) Time-of-day variation defines potential windows for treatment optimization. (**D**) Therapeutic strategies include chronotherapy and circadian stabilization. PD-1, programmed cell death protein 1; PD-L1, programmed death-ligand 1.

Circadian regulation also intersects with pathways involved in angiogenesis, metabolic adaptation, and tumor–microenvironment interactions, further broadening its potential clinical significance. BMAL1-dependent signaling has been implicated in angiogenic regulation and adaptive tumor responses [36], while recent transcriptomic analyses have identified circadian signatures associated with treatment responsiveness and resistance-related phenotypes [42]. These observations suggest that circadian biomarkers may ultimately provide clinically relevant information beyond the assessment of individual clock-gene expression. Potential translational applications of circadian biology in CRC are summarized in Figure 6.

From a translational perspective, circadian biology emerges as a potentially actionable dimension of colorectal cancer management. Circadian pathways influence treatment sensitivity, therapeutic resistance, and tolerance to anticancer therapy, while chronotherapeutic approaches seek to exploit temporal variation in these processes to improve clinical outcomes. Although prospective interventional studies are still required to establish the clinical utility of individualized chronotherapy, the available evidence supports continued investigation of circadian biomarkers and time-informed treatment strategies as promising components of future precision-oncology approaches in CRC. A structured overview of the principal biological domains influenced by circadian dysregulation is provided in Table 1.

Taken together, the evidence synthesized in this review supports the concept that circadian dysregulation orchestrates multiple interconnected biological processes rather than isolated molecular abnormalities, providing the systems-level biological basis for the proposed temporal state framework. This perspective offers an integrated foundation for future mechanistic studies and may facilitate the development of circadian-informed biomarkers and therapeutic strategies in colorectal cancer.

## 4. Discussion

### 4.1. Circadian Dysregulation as a Systems-Level Property of Colorectal Cancer

The central observation emerging from this review is that circadian dysregulation in colorectal cancer cannot be adequately explained by isolated alterations in individual clock genes. Instead, the available evidence indicates coordinated disturbances involving multiple biological processes. Across experimental models and clinical cohorts, abnormalities affecting *PER*, *CRY*, *CLOCK*, *BMAL1*, *ARNTL2*, and *TIMELESS* were consistently associated with altered proliferation, metabolism, epithelial plasticity, immune regulation, and treatment responsiveness. The consistency of these findings across diverse study designs suggests that disruption of temporal regulation represents a recurrent feature of CRC biology rather than a context-specific phenomenon.

This interpretation is consistent with emerging concepts that place biological timing alongside genomic, epigenetic, metabolic, and immune states as an additional dimension of tumor organization [2,4,56]. Circadian regulation functions as an integrative system coordinating multiple adaptive processes that collectively influence tumor behavior, rather than as an isolated signaling pathway. From this perspective, disruption of temporal organization gives rise to the Tumor Temporal State, defined as a multidimensional biological condition characterized by reduced rhythmic coordination across interacting biological systems. This concept provides a unifying perspective for interpreting heterogeneous observations reported across molecular, metabolic, immune, and translational studies while avoiding the assumption that circadian dysregulation itself functions as an independent oncogenic driver. Instead, the available evidence indicates that these biological processes form a temporal regulatory network in which disruption of one component may propagate across multiple functional domains of tumor biology.

### 4.2. Circadian Regulation, Epithelial Plasticity, and Adaptive Fitness

The studies included in this review indicate that circadian regulation influences several fundamental determinants of tumor adaptability. Experimental investigations consistently demonstrated interactions between core clock components and signaling pathways governing proliferation, stemness, epithelial–mesenchymal plasticity, and cellular stress responses. Recent conceptual work further suggests that circadian regulation contributes to the maintenance of cancer stem-cell populations and adaptive tumor fitness, thereby providing a mechanistic link between temporal organization and disease progression [47]. Although the underlying molecular mechanisms differ among experimental systems, disruption of rhythmic regulation consistently favored biological states associated with increased adaptability and more aggressive tumor behavior.

Among the most consistent observations was the recurrent involvement of *TIMELESS*, *BMAL1*, and *ARNTL2* in pathways regulating cellular plasticity and adaptation to environmental stress. Together with earlier studies demonstrating roles for *PER1* in cell-cycle regulation, apoptosis, and DNA-damage responses, these findings indicate that circadian pathways contribute to maintaining genomic stability and epithelial homeostasis [57]. Contemporary reviews have likewise proposed that circadian networks enhance tumor fitness by coordinating responses to environmental fluctuations, treatment-induced stress, and cancer stem-cell maintenance [4,47,58]. The evidence synthesized in the present review extends these concepts to colorectal cancer by demonstrating that circadian regulators participate in multiple biological processes associated with aggressive tumor behavior.

Importantly, the available evidence does not support a simplistic classification of individual clock genes as exclusively oncogenic or tumor suppressive. Instead, their biological effects appear to be highly context dependent, reflecting complex interactions among circadian regulators, oncogenic signaling pathways, metabolic conditions, and the tumor microenvironment. Consequently, circadian dysregulation is best interpreted as disruption of an integrated regulatory network rather than dysfunction of individual genes. This systems-level perspective provides a more comprehensive explanation for the diverse biological consequences of circadian disruption observed across experimental and clinical studies.

### 4.3. The Clock–Microbiota–Immune Axis as a Framework for Metastatic Competence

One of the most consistent observations emerging from the studies included in this review is the convergence of circadian, microbial, metabolic, and immune pathways. Multiple investigations demonstrated that disruption of circadian regulation alters microbiota composition, compromises epithelial barrier function, modifies inflammatory signaling, and reshapes immune-cell activity [7,8,21,25]. Together, these findings support a broader biological framework in which temporal regulation influences tumor progression through coordinated interactions among multiple host physiological systems.

Recent conceptual models have proposed the existence of a clock–microbiota–immune axis integrating environmental signals, microbial metabolites, and immune regulation [4,17]. These observations provide substantial biological support for this concept. Circadian disruption has been associated with altered microbial metabolism, accumulation of immunosuppressive myeloid populations, impaired antitumor immunity, and the establishment of microenvironments that favor tumor progression [21,25]. Importantly, the evidence supporting this proposed axis originates predominantly from experimental models, including genetically modified mice, xenograft systems, and mechanistic cell-culture studies. Although these investigations provide compelling mechanistic evidence linking circadian dysregulation with immune remodeling and metastatic progression, prospective validation in human colorectal cancer remains limited. Accordingly, the proposed mechanisms should currently be regarded as biologically plausible models requiring further clinical confirmation rather than established clinical pathways.

Current findings further indicate that metastatic competence is shaped through coordinated interactions among epithelial plasticity, immune regulation, microbial metabolism, and stromal remodeling. Loss of temporal organization influences several interconnected biological systems simultaneously, thereby amplifying its effects on tumor progression instead of acting through a single dominant metastatic pathway. This systems-level perspective is consistent with the view that metastatic dissemination emerges from dynamic interactions between tumor-intrinsic programs and host-derived biological processes rather than from isolated molecular events.

Despite the growing body of experimental data, the relative contribution of individual components of the proposed clock–microbiota–immune axis to metastatic disease in patients with colorectal cancer remains uncertain. Future prospective studies integrating molecular circadian profiling, microbiome characterization, immune phenotyping, and longitudinal clinical follow-up will be essential to determine the clinical relevance of this framework and to establish whether circadian disruption represents a biologically actionable determinant of metastatic competence.

### 4.4. Clinical Implications and Future Directions for Chronotherapy

Current evidence indicates that circadian regulation influences multiple determinants of response to treatment in colorectal cancer. Instead of acting through a single biological pathway, circadian mechanisms interact with cell-cycle regulation, DNA-damage responses, oxidative-stress adaptation, metabolism, and tumor–microenvironment interactions. As a result, disruption of temporal organization may alter both intrinsic treatment sensitivity and the ability of tumor cells to adapt to treatment-induced stress.

Among the most consistent experimental observations is the role of *BMAL1*-dependent signaling in maintaining treatment sensitivity. Altered *BMAL1* activity affects cell-cycle progression, apoptosis, DNA-damage responses, and metabolic adaptation, thereby modifying susceptibility to anticancer therapies [31,51]. Circadian regulation also contributes to metabolic remodeling through HKDC1-dependent pathways that influence both tumor progression and response to anticancer treatment [18]. Together, these findings indicate that preservation of circadian integrity may enhance treatment efficacy by maintaining coordinated cellular stress responses and limiting adaptive resistance.

The influence of circadian regulation extends beyond malignant cells to the surrounding tumor microenvironment. Interactions between colorectal cancer cells and tumor-associated fibroblasts modify circadian organization, metabolic activity, apoptosis, and resistance to cytotoxic therapy, emphasizing that response to treatment reflects coordinated communication between malignant and stromal compartments rather than tumor cell-intrinsic mechanisms alone [52].

Clinical and translational studies further support the biological relevance of these observations. Associations have been reported between clock-gene expression patterns and responses to chemotherapy or radiochemotherapy, while genetic variation within circadian pathways may contribute to interindividual differences in treatment efficacy and treatment-related toxicity [38,39,40]. Collectively, these findings identify circadian regulation as an important, yet frequently underrecognized, determinant of clinical variability among patients with colorectal cancer.

Recognition that treatment timing may influence clinical outcomes has provided the biological rationale for chronotherapy. Early clinical studies demonstrated that administration of anticancer treatment according to predefined circadian schedules can improve treatment tolerability while maintaining treatment efficacy [22,23,24]. More recent investigations suggest that individual circadian characteristics, including chronotype and sex-related differences, may influence the magnitude of chronotherapeutic benefit and partly explain the variability observed across clinical trials [22,23,24]. Nevertheless, implementation of chronotherapy in routine clinical practice remains limited by the absence of standardized circadian biomarkers and validated approaches for individualized treatment scheduling.

Future studies integrating molecular circadian profiling, chronotype assessment, longitudinal biomarker monitoring, and standardized treatment-timing protocols will be required to determine which patients derive the greatest benefit from circadian-informed treatment strategies. Beyond optimization of treatment timing, circadian biology has the potential to contribute to biomarker development, patient stratification, and precision oncology [22,26,36,42].

Taken together, the available evidence indicates that circadian biology represents a clinically relevant dimension of colorectal cancer management. Although prospective interventional studies remain necessary to establish the clinical utility of individualized chronotherapy, current findings provide a strong rationale for continued investigation of circadian biomarkers and time-informed treatment strategies. Such approaches may ultimately complement existing precision-oncology frameworks by incorporating temporal biology into clinical decision-making.

### 4.5. Interpretation of the Tumor Temporal State Concept

The concept of a Tumor Temporal State emerged from the integration of molecular, experimental, translational, and clinical evidence identified in this systematic review. Rather than functioning as an additional molecular pathway, it is proposed as a conceptual framework describing the extent to which circadian organization is preserved or disrupted across interacting biological systems in colorectal cancer.

The studies synthesized in this review consistently indicate that circadian dysregulation extends beyond alterations in individual clock genes. Instead, disruption of temporal organization is associated with coordinated changes involving metabolism, immune regulation, epithelial plasticity, stromal remodeling, metastatic competence, and treatment responsiveness [4,56]. Integrating these observations within a unified model facilitates interpretation of heterogeneous findings reported across different biological domains and experimental systems while emphasizing their underlying biological relationships.

Importantly, this temporal state should not be regarded as a validated biomarker, diagnostic classifier, or clinical scoring system. Rather, it represents a hypothesis-generating conceptual model designed to organize the current body of evidence and provide a coherent approach for future investigations of multidimensional circadian regulation in colorectal cancer. Prospective validation integrating molecular circadian biomarkers with immune, metabolic, microbiome, and longitudinal clinical data will be required before this concept can be translated into clinical practice.

If validated, the proposed concept may provide a useful basis for integrating circadian biomarkers with other established biological dimensions of colorectal cancer, thereby supporting more comprehensive patient stratification and the future development of chronotherapy-informed precision oncology [22,26,36,42]. Importantly, the Tumor Temporal State is not intended to replace existing pathological or molecular classification systems but rather to complement them by introducing an additional temporal dimension of tumor biology that may improve our understanding of disease heterogeneity and treatment response.

### 4.6. Limitations and Unresolved Questions

Despite growing interest in cancer chronobiology, several important limitations continue to restrict the translation of circadian biology into routine colorectal cancer care. Much of the available evidence originates from experimental models or retrospective clinical studies, whereas standardized and clinically applicable methods for assessing circadian status remain limited. In addition, most investigations rely on single time-point measurements of clock-gene expression, which fail to capture the dynamic nature of circadian regulation and complicate causal interpretation [1,2]. Time of day is also rarely incorporated as a biological variable in clinical studies, limiting assessment of its influence on treatment efficacy, toxicity, and long-term clinical outcomes [22,23,24].

Another important limitation is the considerable methodological diversity of the available literature. The included studies investigated different circadian regulators—including *PER*, *CLOCK*, *CRY*, *BMAL1*, *ARNTL2*, and *TIMELESS*—using diverse experimental systems ranging from human tissues and clinical cohorts to cell cultures, animal models, and chronotherapy studies. Consequently, direct comparison of findings and quantitative synthesis were not methodologically appropriate. Furthermore, most eligible studies reported biologically significant associations, whereas consistently negative findings were uncommon. Although this pattern may reflect the current state of the field, publication bias cannot be excluded and should be considered when interpreting the overall strength of the available evidence.

Several fundamental biological questions also remain unresolved. The relative contributions of tumor-intrinsic circadian clocks and systemic host rhythms—including endocrine, metabolic, microbial, and immune oscillations—have yet to be fully defined [4,17]. Experimental evidence strongly supports the existence of a clock–microbiota–immune axis capable of influencing tumor progression, yet its clinical significance remains to be established in prospective human studies. Similarly, the proposed Tumor Temporal State should currently be regarded as a hypothesis-generating conceptual framework rather than a clinically validated biological entity.

Additional uncertainty arises from the biological heterogeneity of colorectal cancer itself. Circadian regulation may differ according to molecular subtype, anatomical location, disease stage, and treatment context. MSI-high tumors may represent a distinct temporal phenotype, although direct mechanistic evidence remains limited. Likewise, differences in *CRY1* and *CRY2* expression between right- and left-sided colorectal cancers suggest potential spatial variation in circadian regulation, but the biological and clinical implications of these observations remain uncertain [16].

The contribution of circadian dysregulation to treatment resistance also requires further investigation. Emerging evidence links circadian regulators to ferroptosis suppression, pyrimidine metabolism, hypoxia-associated adaptation, immune evasion, and metabolic reprogramming [18,25,48,50,55]. However, the relative importance of these mechanisms in determining treatment failure in patients with colorectal cancer has not yet been established.

Future studies should integrate longitudinal molecular circadian profiling with immune characterization, microbiome analysis, metabolic phenotyping, chronotype assessment, and prospective clinical follow-up. Such multidimensional approaches will be essential to determine the clinical relevance of the proposed clock–microbiota–immune axis, validate the Tumor Temporal State framework, and establish whether circadian biomarkers provide clinically meaningful information beyond individual clock-gene measurements for patient stratification and chronotherapy-guided precision oncology.

## 5. Conclusions

The evidence synthesized in this systematic review indicates circadian dysregulation as a systems-level disturbance that influences metabolism, epithelial plasticity, immune regulation, metastatic competence, and therapeutic responsiveness in colorectal cancer. These alterations extend beyond isolated abnormalities in individual clock genes and instead reflect coordinated disruption of temporal regulation across multiple interconnected biological systems.

Within this context, the proposed temporal state should be regarded as a hypothesis-generating conceptual framework that integrates molecular, metabolic, immune, and clinical observations into a unified model of circadian dysregulation in colorectal cancer. Likewise, the emerging concept of a clock–microbiota–immune axis provides a biologically plausible mechanism linking disruption of temporal organization with tumor progression and metastatic competence, although its clinical relevance requires prospective validation in human colorectal cancer.

From a translational perspective, circadian biology offers promising opportunities for the development of molecular biomarkers, chronotype-informed patient stratification, and biomarker-guided chronotherapy. Future prospective studies integrating longitudinal circadian profiling with immune characterization, microbiome analysis, metabolic phenotyping, and clinical outcome assessment will determine whether circadian biomarkers provide clinically meaningful information beyond existing molecular classifiers and whether restoration of circadian integrity can contribute to precision oncology in colorectal cancer.

Although prospective clinical validation remains necessary, the present review supports the Tumor Temporal State as a hypothesis-generating conceptual model for understanding how circadian dysregulation integrates the metabolic, immune, epithelial, and invasive dimensions of colorectal cancer. This conceptual model provides a foundation for future investigations into circadian biology, precision chronotherapy, and temporally informed precision oncology in colorectal cancer.

## Figures and Tables

**Table 1:** Functional overview of major circadian regulators in colorectal cancer. Summary of the predominant biological functions, principal mechanisms, and clinical relevance of the major circadian regulators identified across the studies included in this review. Detailed characteristics of individual studies are provided in Supplementary Table S1.

Circadian Regulator	Predominant Biological Role	Principal Mechanisms	Clinical/Translational Implications	Key References
*CLOCK*	Predominantly pro-tumor	Migration, invasion, metastatic competence, genomic instability	Advanced stage, lymph-node metastasis, poorer prognosis	[15,28,29]
*BMAL1* (*ARNTL*)	Context-dependent; predominantly protective	Cell-cycle regulation, apoptosis, metabolic homeostasis, chemosensitivity	Improved oxaliplatin response; longer OS/PFS in selected cohorts	[15,17]
*CRY1*	Predominantly pro-tumor	Cell proliferation, migration, tumor growth	Poor prognosis; reduced OS and DFS	[14]
*TIMELESS*	Predominantly pro-tumor	DNA replication/repair, proliferation, migration	Associated with reduced overall survival	[10]
*ARNTL2*	Pro-tumor; therapy resistance	Redox regulation, oxidative-stress adaptation, 5-FU resistance	Potential predictor of treatment response	[11]
period family (*PER1–PER3*)	Predominantly tumor suppressive	DNA repair, apoptosis, cell-cycle control, Wnt regulation	Reduced metastasis and more favorable outcome with preserved expression	[30,31,32]

## Data Availability

No new data were created or analyzed in this study. Data sharing is not applicable to this article.

## Supplementary Materials

The following supporting information can be downloaded at https://www.mdpi.com/article/10.3390/ijms27146164/s1. Reference [59] is cited in the Supplementary Materials.

## Abbreviations

AKT—protein kinase B. ARNTL2—Aryl hydrocarbon receptor nuclear translocator-like protein 2. BMAL1—Brain and muscle ARNT-like protein 1. CLOCK—Circadian locomotor output cycles kaput. CRC—Colorectal cancer. CRY—Cryptochrome. CRY1—Cryptochrome circadian regulator 1. DFS—disease-free survival. DNA—deoxyribonucleic acid. 5-FU—5-fluorouracil. G2/M—Gap 2/Mitosis phase of the cell cycle. HKDC1—Hexokinase domain-containing protein 1. MSI—Microsatellite instability. mTOR—mechanistic target of rapamycin. NDRG2—N-myc downstream-regulated gene 2. NK cells—Natural killer cells. P21—Cyclin-dependent kinase inhibitor 1A (CDKN1A). P53—Tumor protein p53. PER—Period circadian regulator. PER1–3—Period circadian regulators 1–3. PFS—progression-free survival. P-gp—P-glycoprotein. REV-ERBα/β—Nuclear receptor subfamily 1 group D members alpha and beta. PRISMA—Preferred Reporting Items for Systematic Reviews and Meta-Analyses. RORα/β—Retinoic acid receptor-related orphan receptor alpha and beta. SLC7A11—Solute carrier family 7 member 11. Wnt/β-catenin—Wnt signaling pathway mediated by β-catenin.
