# Supplementary material for: Circadian Disruption as a Determinant of the Tumor Temporal State in Colorectal Cancer: A PRISMA-Based Systematic Review Integrating Metabolism, Immunity, and Metastasis
Source: Int J Mol Sci. 2026 Jul 10;27(14):6164. doi: 10.3390/ijms27146164 (PMC13410881; doi:10.3390/ijms27146164)
Supplement: Supplementary file 1 [file ijms-27-06164-s001.zip › ijms-4393036-supplementary.pdf]

Supplementary Table S1. Characteristics of included primary studies

Author	Year	Study type	Model	Circadian target	Main findings
Section A. Human CRC studies (n = 20)					
Mostafaie	2009	Human tissue/translational study	Primary colorectal tumors and adjacent normal mucosa	PER1, PER2, CLOCK; estrogen receptor β	PER1 and estrogen receptor β were downregulated in colorectal tumors, with a significant correlation between both transcripts and sex-specific differences in PER1/CLOCK expression.
Alhopuro	2010	Human genetic/tissue study with functional context	Colorectal tumors; CRC cell-line functional validation	CLOCK; PER1	Somatic CLOCK alterations were identified in CRC, supporting disruption of core clock circuitry as a tumor-associated molecular event.
Zhou	2012	Clinical genetic association study	Patients with resected colorectal cancer	CLOCK, BMAL1/ARNTL, NPAS2 and circadian positive-feedback genes	Functional polymorphisms in circadian positive-feedback genes were associated with clinical outcome after CRC resection.
Mazzoccoli	2012	Human tissue/translational biomarker study	Human CRC tissue and clinicopathologic correlations	ARNTL2; SERPINE1	ARNTL2 and SERPINE1 were proposed as biomarkers linked to tumor aggressiveness in colorectal cancer.
Wang X	2012	Human tissue/prognostic study	Colon cancer tissues and clinical samples	PER3	Reduced PER3 expression was associated with colon cancer incidence and development.
Wang L	2013	Human expression study	Human colorectal carcinoma samples	CLOCK	CLOCK expression was altered/upregulated in human colorectal carcinoma, linking CLOCK dysregulation with CRC biology.
Yu	2013	Human tissue/prognostic study with experimental validation	CRC patients; human CRC tissue; CRC cell lines	CRY1	CRY1 overexpression correlated with tumor progression and poor prognosis in patients with CRC.
Wang Y	2015	Human tissue expression study	Chinese colon cancer tissues and adjacent mucosa	PER1; estrogen receptor β	PER1 and estrogen receptor β were downregulated in colon cancers, supporting loss of clock-associated transcriptional organization.
Lu	2015	Clinical/tissue treatment-response study	Rectal cancer patients treated with neoadjuvant chemoradiation; tumor tissue; CRC models	CLOCK, BMAL1, PER1, PER2, CRY1, CRY2	Circadian gene-expression patterns predicted response to neoadjuvant chemoradiation therapy in rectal cancer.
Garufi	2016	Clinical genetic association study	Metastatic CRC patients	Clock-gene SNPs; miRNAs targeting clock genes	Sex-specific effects of clock-gene SNPs and regulatory miRNAs were associated with clinical features/outcomes in metastatic CRC.
Huisman	2016	Human paired tissue study	Primary CRC and colorectal liver metastases from the same patients	Core clock genes; cell-cycle genes	Clock-gene expression was disrupted in CRC and matched colorectal liver metastases, suggesting altered temporal regulation during metastatic progression.
Mazzoccoli	2016	Human tissue/translational study	CRC patients; tumor and adjacent tissue; CRC cell models	CRY1, CRY2; ARNTL/BMAL1	Cryptochrome genes were deregulated in human CRC, implicating altered negative-arm clock signaling in tumor biology.

Wang Y	2017	Human tissue and experimental study	Human CRC tissue; CRC cell lines; animal model	CLOCK	Upregulation of CLOCK was associated with invasive tumor behavior and metastatic progression in CRC.
Hasakova	2018	Human tissue expression study	CRC and adjacent tissues	CRY1, CRY2	CRY1 and CRY2 expression patterns differed between tumor and adjacent tissue and varied according to tumor location.
Hasakova	2018	Human clinical/tissue prognostic study	CRC patients/cohort; human CRC tissue; animal-model context	CLOCK, PER2, CRY1, CRY2, TIM	Clock-gene expression showed sex-dependent associations with survival in patients with CRC.
Burgermeister	2019	Clinical-translational treatment-response study	CRC patients; CRC cell lines; xenograft models	BMAL1/ARNTL; VEGFA	BMAL1/ARNTL was associated with bevacizumab resistance through regulation of VEGFA.
Herichova	2019	Human tissue/sex-stratified expression study	Human CRC tissues	PER2, CRY1, CRY2; estrogen receptor β; VEGF-A	Sex-dependent ERβ regulation in CRC was linked to clock-gene and VEGF-A expression patterns.
Cao	2021	Human tissue and mechanistic translational study	Human CRC tissue; CRC cell lines; mouse models	TIMELESS	H3K27ac-mediated activation of TIMELESS promoted CRC tumorigenesis, migration, and invasion through MYH9-associated mechanisms.
Krugluger	2007	Human translational study	Human colorectal cancer tissues	PER1, DPD	Reduced PER1 and dihydropyrimidine dehydrogenase (DPD) expression correlated with high-grade colorectal tumors, suggesting early involvement of circadian dysregulation in CRC progression.
Tao	2025	Computational human transcriptomic study	CRC scRNA-seq and bulk RNA-seq datasets	Circadian rhythm-related genes	Circadian rhythm-disruption gene signatures were associated with prognosis, drug resistance, immune pathways, and treatment-response features in CRC datasets.
Section B. Experimental CRC studies (n = 19)					
Zeng	2014	Experimental and translational treatment-sensitivity study	CRC cells; xenograft/animal model; oxaliplatin context	BMAL1	BMAL1 overexpression increased sensitivity to oxaliplatin in CRC models.
Zeng	2010	Experimental in vitro/in vivo study	CRC cell lines and animal models	BMAL1, PER1, PER2, PER3	BMAL1 manipulation altered tumor growth and anticancer drug activity, supporting clock-dependent control of treatment sensitivity.
Sakamoto	2015	Experimental cell-line study	Human colon cancer cells	CLOCK, BMAL1	Overexpression of CLOCK and BMAL1 inhibited entry into S phase in human colon cancer cells.
Hong	2014	Experimental mechanistic study	CRC cell lines	PER3; miR-103	PER3 acted as a suppressive target of miR-103 in colorectal cancer cells.
Zhang F	2017	Experimental cancer stem-like cell study	CRC stem-like cell models	PER3; Notch; β-catenin	PER3 overexpression inhibited self-renewal capability and chemoresistance of CRC stem-like cells through Notch and β-catenin signaling.

Fuhr	2018	Experimental metabolic and treatment-response study	CRC cell lines; animal models; metabolic phenotyping	Circadian clock; HKDC1	The circadian clock regulated metabolic phenotype rewiring via HKDC1 and modulated tumor progression and drug response in CRC.
Basti	2022	Experimental in vitro/zebrafish xenograft study	CRC cell lines; zebrafish xenograft model	NR1D1, BMAL1, PER2	NR1D1/core-clock disruption altered cell motility, invasion, proliferation, apoptosis, and dissemination-associated phenotypes.
Bishehsari	2020	Experimental carcinogenesis study	Alcohol-associated colon carcinogenesis models; intestinal/circadian models	PER2; circadian disruption	Abnormal eating patterns caused circadian disruption and promoted alcohol-associated colon carcinogenesis.
Zhang Y	2020	Experimental cell-fate study	Colon carcinoma cell lines	BMAL1; AKT/mTOR; p53/p21	BMAL1 knockdown triggered different colon carcinoma cell fates by altering the balance between AKT/mTOR and p53/p21 signaling pathways.
Zhang Y	2021	Experimental cell-line study	Colon carcinoma cell lines	BMAL1	BMAL1 knockdown shifted epithelial–mesenchymal balance toward epithelial properties and decreased chemoresistance.
Fuhr	2019	Experimental tumor–stroma interaction study	HCT116 cells; normal fibroblasts; tumor-associated fibroblasts	BMAL1; circadian clock; tumor microenvironment	Interactions between colorectal cancer cells and tumor-associated fibroblasts disrupted circadian organization, altered metabolic activity, reduced apoptosis, and increased resistance to cytotoxic therapy.
Olejárová	2022	Experimental cell-line study	DLD1 colorectal cancer cells	BMAL1, CLOCK, CRY, PER; miR-34a; SIRT1	A 2.4 GHz electromagnetic field influenced circadian-oscillator responses to miR-34a/SIRT1-mediated regulation in DLD1 cells, representing an exploratory model of clock-gene modulation.
Moravčík	2023	Experimental cell-line study	DLD1 and LoVo human CRC cell lines	miR-34a; PER2; BMAL1; REV-ERBα; CLOCK; SIRT1	miR-34a altered expression of clock and clock-controlled genes in CRC cells, with p53- and estradiol-dependent context specificity.
Fellows	2024	Experimental intestinal-clock study	Animal CRC models; intestinal barrier/microbiota model	Intestinal circadian clock	Disruption of the intestinal clock drove dysbiosis and impaired barrier function in CRC models.
Fortin	2024	Experimental immunotherapy-timing study	Genetic CRC mouse model; single-cell profiling; immune checkpoint blockade	Circadian clock; PD-L1; MDSCs	Circadian control of tumor immunosuppression gated myeloid suppressor-cell abundance and influenced efficacy of immune checkpoint blockade.
Ran	2025	Experimental treatment-resistance study	CRC cells; human tissue validation	HIF-1α/BMAL1/ALDOC axis	Hypoxia regulated glycolysis through the HIF-1α/BMAL1/ALDOC axis and reduced oxaliplatin sensitivity in CRC.
Yang	2025	Experimental chemoresistance study	Colon cancer cells; animal models; human datasets/tissues	ARNTL2; SLC7A11; ferroptosis; melatonin	ARNTL2 enhanced 5-FU resistance by upregulating SLC7A11 and suppressing ferroptosis; melatonin reduced ARNTL2 signaling.

Li	2026	Experimental translational treatment-response study	Human CRC tissue; animal models	CLOCK stability; NDRG2; oxaliplatin response	NDRG2 orchestrated circadian clock stability, suppressed tumorigenesis, and potentiated oxaliplatin response in CRC.
Liu	2024	Experimental/translational metastasis study	CRC patients; CRC cell lines; mouse metastasis models; gut microbiota/metabolites	Circadian clock; microbiota; MDSCs; taurocholic acid	Circadian disruption promoted CRC lung metastasis through microbiota/metabolite-driven accumulation of myeloid-derived suppressor cells.
Section C. Chronotherapy studies (n = 4)					
Mormont	2002	Clinical chronobiology study	Patients with metastatic CRC and good performance status	Marker rhythms of circadian system function	Circadian-system marker rhythms were characterized in metastatic CRC patients, supporting clinical assessment of circadian-system function
Niu	2022	Genome-wide CRISPR chronochemotherapy study	CRC cell lines; animal models	5-FU chronochemotherapy; pyrimidine metabolism; circadian clock	Genome-wide CRISPR screening identified pyrimidine metabolic reprogramming as a determinant of 5-FU chronochemotherapy in CRC.
Dulong	2015	In vitro chronopharmacology and mathematical modeling	Cancer chronopharmacology models	Circadian determinants of chronotherapy	In vitro chronopharmacology and mathematical modeling identified circadian determinants relevant to cancer chronotherapy.
Ballesta	2011	Experimental/computational chronotherapy optimization study	CRC cell lines; animal models; irinotecan delivery modeling	CLOCK, BMAL1, PER2; chronomodulated irinotecan	Combined experimental and mathematical modeling supported molecular optimization of irinotecan circadian delivery.

Supplementary Table S2. Evidence classification and methodological appraisal of included studies. Methodological appraisal was performed according to study design. Observational clinical studies were assessed using criteria adapted from the Newcastle–Ottawa Scale (selection of study population, comparability of groups, ascertainment of outcomes, and overall methodological quality). Experimental cell-culture and animal studies were qualitatively evaluated with respect to experimental design, biological validation, mechanistic depth, reproducibility, and translational relevance. Chronotherapy studies were additionally assessed according to study design, timing of therapeutic intervention, clinical endpoints, and potential applicability to clinical practice.

Author	Year	Study category	Evidence type	Primary domain	Methodological appraisal
Section A. Human CRC studies (n = 20)					
Mostafaie	2009	Human CRC studies	Human observational / translational	PER1/ERβ downregulation	Moderate
Alhopuro	2010	Human CRC studies	Human observational / translational	CLOCK alterations	Moderate
Zhou	2012	Human CRC studies	Clinical genetic association	Circadian polymorphisms / prognosis	Moderate
Mazzoccoli	2012	Human CRC studies	Human observational / translational	ARNTL2/SERPINE1 aggressiveness biomarkers	Moderate
Wang X	2012	Human CRC studies	Human observational / translational	PER3 expression	Moderate
Wang L	2013	Human CRC studies	Human observational / translational	CLOCK expression	Moderate
Yu	2013	Human CRC studies	Human observational / translational	CRY1 prognosis and function	Moderate
Wang Y	2015	Human CRC studies	Human observational / translational	PER1/ERβ expression	Moderate
Lu	2015	Human CRC studies	Clinical/translational treatment-response	Neoadjuvant chemoradiation response	Moderate

Garufi	2016	Human CRC studies	Clinical genetic association	Clock-gene SNPs / miRNAs	Moderate
Huisman	2016	Human CRC studies	Human observational / translational	Clock disruption in liver metastases	Moderate
Mazzoccoli	2016	Human CRC studies	Human observational / translational	CRY1/CRY2 deregulation	Moderate
Wang Y	2017	Human CRC studies	Translational mechanistic	CLOCK-associated invasion and metastatic behavior	High
Hasakova	2018	Human CRC studies	Human observational / translational	CRY1/CRY2 expression and prognosis/location	Moderate
Hasakova	2018	Human CRC studies	Human observational / translational	Sex-dependent clock-gene expression and survival	Moderate
Burgermeister	2019	Human CRC studies	Clinical/translational treatment-response	BMAL1-associated VEGFA-mediated treatment resistance	Moderate
Herichova	2019	Human CRC studies	Human observational / translational	ERβ-clock-VEGF-A interaction	Moderate
Cao	2021	Human CRC studies	Translational mechanistic	TIMELESS/MYH9-mediated tumorigenesis, migration and invasion	High
Krugluger	2007	Human CRC studies	Human translational	PER1 dysregulation and tumor progression	Moderate
Tao	2025	Human CRC studies	Computational / transcriptomic	Circadian transcriptomic signatures	Exploratory
Section B. Experimental CRC studies (n = 19)					
Zeng	2014	Experimental CRC studies	Experimental therapeutic-sensitivity study	BMAL1 and oxaliplatin sensitivity	High
Liu	2024	Experimental CRC studies	Translational mechanistic	Clock-microbiota-MDSC metastasis axis	High
Zeng	2010	Experimental CRC studies	Experimental mechanistic	BMAL1 and anticancer drug activity	High
Sakamoto	2015	Experimental CRC studies	Experimental mechanistic	CLOCK/BMAL1 and cell-cycle entry	High
Hong	2014	Experimental CRC studies	Experimental mechanistic	miR-103-PER3 tumor suppression	High
Zhang F	2017	Experimental CRC studies	Experimental mechanistic	PER3 / Notch / β-catenin stem-like cells	High
Fuhr	2018	Experimental CRC studies	Experimental mechanistic	HKDC1 metabolic rewiring / drug response	High
Basti	2022	Experimental CRC studies	Experimental mechanistic	NR1D1/core-clock invasion and dissemination-associated phenotypes	High
Bishehsari	2020	Experimental CRC studies	Experimental mechanistic	Eating-pattern disruption / carcinogenesis	High
Zhang Y	2020	Experimental CRC studies	Experimental mechanistic	BMAL1 / AKT-mTOR / p53-p21 cell fate	High
Zhang Y	2021	Experimental CRC studies	Experimental mechanistic	BMAL1 and EMT/chemoresistance	High
Fuhr	2019	Experimental CRC studies	Experimental mechanistic	Tumor-stroma clockwork and chemoresistance	High
Olejárová	2022	Experimental CRC studies	Experimental mechanistic	miR-34a / SIRT1 / oscillator response	Moderate
Moravčík	2023	Experimental CRC studies	Experimental mechanistic	miR-34a-clock gene regulation	Moderate
Fellows	2024	Experimental CRC studies	Experimental mechanistic	Intestinal clock / dysbiosis / barrier dysfunction	High
Fortin	2024	Experimental CRC studies	Experimental mechanistic	Circadian immunosuppression / ICB efficacy	High
Ran	2025	Experimental CRC studies	Experimental mechanistic	HIF-1α/BMAL1/ALDOC oxaliplatin resistance	High

Yang	2025	Experimental CRC studies	Experimental mechanistic	ARNTL2/SLC7A11 ferroptosis and 5-FU resistance	High
Li	2026	Experimental CRC studies	Translational mechanistic	NDRG2 / clock stability / oxaliplatin response	High
Section C. Chronotherapy studies (n = 4)					
Mormont	2002	Chronotherapy studies	Clinical chronobiology	Circadian marker rhythms in metastatic CRC	Moderate
Niu	2022	Chronotherapy studies	Functional screening / chronotherapy	Pyrimidine metabolism and 5-FU chronochemotherapy	High
Dulong	2015	Chronotherapy studies	Experimental/computational chronotherapy	Circadian determinants of chronotherapy	High
Ballesta	2011	Chronotherapy studies	Computational / hypothesis-generating	Irinotecan circadian delivery optimization	Exploratory

Supplementary Table S3. Complete database-specific search strategies and study identification process used for the systematic review

Database-specific electronic search strategies

Database	Complete search strategy
PubMed/MEDLINE	("colorectal cancer"[Title/Abstract] OR "colon cancer"[Title/Abstract] OR "rectal cancer"[Title/Abstract]) AND ("circadian rhythm"[Title/Abstract] OR "circadian clock"[Title/Abstract] OR chronobiology[Title/Abstract] OR chronotherapy[Title/Abstract] OR CLOCK[Title/Abstract] OR BMAL1[Title/Abstract] OR ARNTL[Title/Abstract] OR ARNTL2[Title/Abstract] OR PER1[Title/Abstract] OR PER2[Title/Abstract] OR PER3[Title/Abstract] OR CRY1[Title/Abstract] OR CRY2[Title/Abstract] OR TIMELESS[Title/Abstract])
Scopus	TITLE-ABS-KEY ("colorectal cancer" OR "colon cancer" OR "rectal cancer") AND TITLE-ABS-KEY ("circadian rhythm" OR "circadian clock" OR chronobiology OR chronotherapy OR CLOCK OR BMAL1 OR ARNTL OR ARNTL2 OR PER1 OR PER2 OR PER3 OR CRY1 OR CRY2 OR TIMELESS)
Web of Science Core Collection	TS=("colorectal cancer" OR "colon cancer" OR "rectal cancer") AND TS=("circadian rhythm" OR "circadian clock" OR chronobiology OR chronotherapy OR CLOCK OR BMAL1 OR ARNTL OR ARNTL2 OR PER1 OR PER2 OR PER3 OR CRY1 OR CRY2 OR TIMELESS)

Search source	Year/period	Study category	Search framework	Primary domain	Contribution to evidence synthesis
Section A. Electronic database searches (n = 3)					
PubMed/MEDLINE	Inception–May 2026	Electronic database search	Controlled vocabulary and free-text search	Clinical, translational and experimental CRC circadian literature	Core biomedical evidence source contributing substantially to the final review dataset.
Scopus	Inception–May 2026	Electronic database search	Database-adapted keyword strategy	Multidisciplinary biomedical and translational literature	Expanded retrieval sensitivity and multidisciplinary evidence coverage.
Web of Science Core Collection	Inception–May 2026	Electronic database search	Topic-based search strategy	Citation-indexed biological and clinical literature	Citation-indexed supporting evidence source.
Section B. Supplementary identification procedures (n = 3)					
Reference-list screening	May 2026	Supplementary search procedure	Manual bibliography review	Studies not captured electronically	Improved completeness of evidence retrieval.
Citation tracking	May 2026	Supplementary search procedure	Forward citation assessment	Influential circadian CRC publications	Verification of evidence saturation and retrieval completeness.
Duplicate assessment	Entire process	Data management procedure	Automated and manual deduplication	Construction of unique screening dataset	Final screening dataset established.

Section C. Study selection and eligibility assessment (n = 4)					
Title screening	Screening phase	Eligibility assessment	Title-level relevance assessment	CRC and circadian biology relevance	Removal of clearly non-relevant reports.
Abstract screening	Screening phase	Eligibility assessment	Abstract-level review	Methodological and thematic relevance	Construction of candidate evidence dataset.
Full-text assessment	Eligibility phase	Eligibility assessment	Application of predefined criteria	Methodological and thematic eligibility	Identification of eligible studies.
Qualitative synthesis	Final phase	Evidence synthesis	Structured extraction and appraisal	Integrated evidence synthesis	43 studies included in final review dataset.

Searches were performed from database inception through May 2026. No restrictions regarding publication year were applied. Only peer-reviewed articles published in English were considered. Reference lists of eligible studies were manually screened to identify additional relevant publications.

A total of 1,338 records were identified through database searching; 315 duplicates were removed; 1,023 records underwent title and abstract screening; 518 reports were assessed in full text; and 43 studies met eligibility criteria and were included in the final evidence synthesis.

Supplementary Table S4. Representative examples of full-text studies excluded after eligibility assessment, together with the primary reason for exclusion

Author	Year	Study type	Title / main topic	Exclusion category	Reason for exclusion
Section A. Non-primary, review, editorial, bibliometric, case-report, or retracted records (representative examples)					
Kattner	2025	Editorial	Rhythms under tension: Circadian clocks in an Unsynced Society.	Non-primary publication	Editorial perspective; no original CRC-specific experimental, translational, or clinical data.
Fernandez	2019	Editorial	Longer Intervals from Neoadjuvant Therapy to Rectal Cancer Surgery: The Clock is Ticking.	Non-primary publication	Editorial commentary; the term clock refers to timing interval rather than circadian biology.
Chen	2025	Bibliometric analysis	Deciphering the circadian rhythm in colorectal cancer: a bibliometric analysis of research landscape and trends.	Secondary literature / bibliometric record	Bibliometric mapping study; not original biological or clinical evidence for extraction.
Della-Morte	2019	Letter	Deregulation of the circadian clock machinery: A novel biomarker for anti-angiogenic drug resistance in colorectal cancer.	Non-primary publication	Letter/commentary format; not an independent primary study for evidence extraction.
Yuan	2019	Meta-analysis / computational review	Identification and meta-analysis of copy number variation-driven circadian clock genes for colorectal cancer.	Secondary / signature-focused evidence	Meta-analytic/signature-based record rather than directly extractable primary circadian CRC evidence.
Du	2025	Case report	A new way to remedy anastomotic leakage after TaTME: A case report.	Case report / clinically unrelated topic	Case report focused on surgical complication management; outside review objectives.

Zhang	2022	Retracted article	RUNX3-regulated circRNA METTL3 inhibits colorectal cancer proliferation and metastasis via miR-107/PER3 axis.	Retracted publication	Retracted publication; excluded from evidence synthesis.
Zhang	2024	Retraction notice	Retraction Note: RUNX3-regulated circRNA METTL3 inhibits colorectal cancer proliferation and metastasis via miR-107/PER3 axis.	Retraction notice	Retraction notice; not eligible as primary evidence.
Section B. Non-CRC, clinically unrelated, or query-artifact records (representative examples)					
Hases	2022	Review chapter / molecular overview	ERbeta and Inflammation.	No circadian endpoint	Inflammation/ERbeta-focused publication; no circadian-clock or chronotherapy endpoint relevant to CRC synthesis.
Helderman	2025	Human genetic study	Clinical syndromes linked to biallelic germline variants in MCM8 and MCM9.	Non-CRC / clinically unrelated	Genetic syndrome study not focused on CRC circadian biology.
Morena	2024	Preclinical transcriptomic study	Transcriptional analysis of cancer cachexia: conserved and unique features across preclinical models and biological sex.	Non-CRC or mixed-model evidence	Cancer-cachexia transcriptomic study without extractable CRC circadian evidence.
Beppu	2023	Surgical technique report	Transanal Minimally Invasive Surgical Approach to Total Pelvic Exenteration.	Irrelevant clinical/surgical topic	Surgical approach study captured by colorectal terminology but unrelated to circadian regulation.
Daly	2025	Clinical colonoscopy study	Is Cognitive Impairment Associated with Inadequate Bowel Preparation for Colonoscopy?	Irrelevant clinical topic	Colonoscopy/bowel-preparation study; no circadian or tumor-clock endpoint.
Daly	2022	Clinical screening study	Clock-Drawing Test as a Screening Tool for Cognitive Impairment Associated With Fecal Immunochemical Test Collection Errors.	Query artifact	Clock refers to a cognitive screening test, not circadian-clock biology.
Graham	2020	Clinical conceptual article	No Barrett's-No Cancer: A Proposed New Paradigm for Prevention of Esophageal Adenocarcinoma.	Non-CRC malignancy / disease scope mismatch	Esophageal adenocarcinoma prevention topic; outside CRC eligibility criteria.
Hammon	2013	Clinical case report	Fat Necrosis of the Breast Following Folinic Acid Extravasation.	Clinically unrelated topic	Breast/folinic-acid extravasation report; no CRC circadian biology.
Section C. Aging-clock, indirect computational, and no-circadian-endpoint records (representative examples)					
Ye	2025	Human epigenetic study	Accelerating epigenetic age in right-sided colon cancer predicts a favorable outcome.	Epigenetic aging-clock study	Addresses epigenetic age acceleration rather than circadian-clock regulation.

Durso	2017	Human biomarker study	Acceleration of leukocytes' epigenetic age as an early tumor and sex-specific marker of breast and colorectal cancer.	Epigenetic aging-clock study	Biological aging-clock focus; not a circadian-clock study.
Alexander	2017	Case-control methylation study	Case-control study of candidate gene methylation and adenomatous polyp formation.	No eligible CRC circadian endpoint	Adenomatous-polyp methylation study; no CRC circadian-clock outcome for extraction.
Widayati	2023	Computational/epigenetic study	Open access-enabled evaluation of epigenetic age acceleration in colorectal cancer and development of a classifier with diagnostic potential.	Epigenetic aging-clock / classifier study	Epigenetic age-classifier work rather than circadian-clock biology.
Sun	2025	Human epigenetic epidemiology study	Accelerated biological aging and its hallmarks in DNA methylation drive the association between unhealthy lifestyles and the onset of colorectal cancer.	Biological aging-clock study	Focuses on DNA-methylation aging/hallmarks rather than circadian disruption.
Qiu	2019	Mixed-cancer molecular study	Research on circadian clock genes in common abdominal malignant tumors.	Mixed/pan-cancer evidence	Abdominal malignancy study without clearly extractable CRC-specific primary evidence for the review.
Keerthana	2023	Computational expression analysis	Identification of potential circadian genes and associated pathways in colorectal cancer progression and prognosis using microarray gene expression analysis.	Computational/signature-only evidence	Indirect microarray/signature analysis; insufficient primary biological or clinical validation for S1 extraction.
Pan	2026	Clinical sleep/neurocognitive study	Multimodal brain-gut-sleep phenotypes predict delirium, long-term cognitive decline, and survival after colorectal cancer surgery.	Indirect clinical sleep phenotype	Postoperative sleep/neurocognitive phenotype study; not tumor circadian-clock biology.
Soares	2021	Human germline/somatic sequencing study	Germline and Somatic Whole-Exome Sequencing Identifies New Candidate Genes Involved in Familial Predisposition to Serrated Polyposis Syndrome.	No circadian endpoint	Serrated polyposis genetics study without circadian-clock or chronotherapy endpoint.

Note. Representative examples are shown to document full-text/detailed eligibility exclusions. Exclusion categories were assigned hierarchically to one dominant reason to avoid double counting. The complete eligibility audit comprised 475 excluded reports: secondary or non-primary literature (n = 138), non-CRC or non-extractable mixed-cancer evidence (n = 126), no circadian-clock/chronotherapy endpoint (n = 101), epigenetic or biological aging-clock studies (n = 38), computational or signature-only evidence (n = 37), irrelevant clinical/surgical topics (n = 20), overlapping datasets (n = 9), and insufficient or abstract-only reporting (n = 7).
Abbreviations: CRC, colorectal cancer; TTME, transanal total mesorectal excision.

Supplementary Table S5. PRISMA 2020 Checklist

Section and Topic	Item #	Checklist item	Location where item is reported
TITLE			
Title	1	Identify the report as a systematic review.	Title page
ABSTRACT			
Abstract	2	See the PRISMA 2020 for Abstracts checklist.	Abstract
INTRODUCTION			

Section and Topic	Item #	Checklist item	Location where item is reported
Rationale	3	Describe the rationale for the review in the context of existing knowledge.	Introduction, paragraphs 1–5
Objectives	4	Provide an explicit statement of the objective(s) or question(s) the review addresses.	End of Introduction
METHODS			
Eligibility criteria	5	Specify the inclusion and exclusion criteria for the review and how studies were grouped for the syntheses.	Methods 2.2
Information sources	6	Specify all databases, registers, websites, organisations, reference lists and other sources searched or consulted to identify studies. Specify the date when each source was last searched or consulted.	Methods 2.1; Supplementary Table S3
Search strategy	7	Present the full search strategies for all databases, registers and websites, including any filters and limits used.	Methods 2.1; Supplementary Table S3
Selection process	8	Specify the methods used to decide whether a study met the inclusion criteria of the review, including how many reviewers screened each record and each report retrieved, whether they worked independently, and if applicable, details of automation tools used in the process.	Methods 2.3; Figure 1
Data collection process	9	Specify the methods used to collect data from reports, including how many reviewers collected data from each report, whether they worked independently, any processes for obtaining or confirming data from study investigators, and if applicable, details of automation tools used in the process.	Methods 2.3
Data items	10a	List and define all outcomes for which data were sought. Specify whether all results that were compatible with each outcome domain in each study were sought (e.g. for all measures, time points, analyses), and if not, the methods used to decide which results to collect.	Methods 2.3
	10b	List and define all other variables for which data were sought (e.g. participant and intervention characteristics, funding sources). Describe any assumptions made about any missing or unclear information.	Methods 2.3
Study risk of bias assessment	11	Specify the methods used to assess risk of bias in the included studies, including details of the tool(s) used, how many reviewers assessed each study and whether they worked independently, and if applicable, details of automation tools used in the process.	Methods 2.4; Supplementary Table S2
Effect measures	12	Specify for each outcome the effect measure(s) (e.g. risk ratio, mean difference) used in the synthesis or presentation of results.	N/A (no meta-analysis)
Synthesis methods	13a	Describe the processes used to decide which studies were eligible for each synthesis (e.g. tabulating the study intervention characteristics and comparing against the planned groups for each synthesis (item #5)).	Methods 2.3
	13b	Describe any methods required to prepare the data for presentation or synthesis, such as handling of missing summary statistics, or data conversions.	N/A
	13c	Describe any methods used to tabulate or visually display results of individual studies and syntheses.	Methods 2.3; Supplementary Tables S1–S2; Figures 2–6
	13d	Describe any methods used to synthesize results and provide a rationale for the choice(s). If meta-analysis was performed, describe the model(s), method(s) to identify the presence and extent of statistical heterogeneity, and software package(s) used.	Methods 2.4
	13e	Describe any methods used to explore possible causes of heterogeneity among study results (e.g. subgroup analysis, meta-regression).	N/A
	13f	Describe any sensitivity analyses conducted to assess robustness of the synthesized results.	N/A
Reporting bias assessment	14	Describe any methods used to assess risk of bias due to missing results in a synthesis (arising from reporting biases).	*Not performed because this review represents a*

Section and Topic	Item #	Checklist item	Location where item is reported
			qualitative evidence synthesis and no quantitative meta-analysis was conducted.
Certainty assessment	15	Describe any methods used to assess certainty (or confidence) in the body of evidence for an outcome.	Formal certainty-of-evidence assessment (e.g., GRADE) was not applicable because no quantitative meta-analysis was performed.
RESULTS			
Study selection	16a	Describe the results of the search and selection process, from the number of records identified in the search to the number of studies included in the review, ideally using a flow diagram.	Methods 2.3; Figure 1
	16b	Cite studies that might appear to meet the inclusion criteria, but which were excluded, and explain why they were excluded.	Supplementary Table S4 (Representative full-text studies excluded after eligibility assessment)
Study characteristics	17	Cite each included study and present its characteristics.	Supplementary Table S1
Risk of bias in studies	18	Present assessments of risk of bias for each included study.	Supplementary Table S2
Results of individual studies	19	For all outcomes, present, for each study: (a) summary statistics for each group (where appropriate) and (b) an effect estimate and its precision (e.g. confidence/credible interval), ideally using structured tables or plots.	Results 3.1–3.4; Supplementary Table S1
Results of syntheses	20a	For each synthesis, briefly summarise the characteristics and risk of bias among contributing studies.	Results 3.2–3.4
	20b	Present results of all statistical syntheses conducted. If meta-analysis was done, present for each the summary estimate and its precision (e.g. confidence/credible interval) and measures of statistical heterogeneity. If comparing groups, describe the direction of the effect.	N/A
	20c	Present results of all investigations of possible causes of heterogeneity among study results.	N/A
	20d	Present results of all sensitivity analyses conducted to assess the robustness of the synthesized results.	N/A
Reporting biases	21	Present assessments of risk of bias due to missing results (arising from reporting biases) for each synthesis assessed.	Not assessed
Certainty of evidence	22	Present assessments of certainty (or confidence) in the body of evidence for each outcome assessed.	Formal GRADE assessment was not applicable because no quantitative meta-analysis was performed.
DISCUSSION			
Discussion	23a	Provide a general interpretation of the results in the context of other evidence.	Discussion 4.1–4.5
	23b	Discuss any limitations of the evidence included in the review.	Discussion 4.6
	23c	Discuss any limitations of the review processes used.	Discussion 4.6
	23d	Discuss implications of the results for practice, policy, and future research.	Discussion 4.4–4.5
OTHER INFORMATION			
Registration and protocol	24a	Provide registration information for the review, including register name and registration number, or state that the review was not registered.	Methods 2.1

Section and Topic	Item #	Checklist item	Location where item is reported
	24b	Indicate where the review protocol can be accessed, or state that a protocol was not prepared.	Methods 2.1
	24c	Describe and explain any amendments to information provided at registration or in the protocol.	Not applicable
Support	25	Describe sources of financial or non-financial support for the review, and the role of the funders or sponsors in the review.	Funding statement
Competing interests	26	Declare any competing interests of review authors.	Conflicts of Interest statement
Availability of data, code and other materials	27	Report which of the following are publicly available and where they can be found: template data collection forms; data extracted from included studies; data used for all analyses; analytic code; any other materials used in the review.	Data Availability Statement

From: Page MJ, McKenzie JE, Bossuyt PM, Boutron I, Hoffmann TC, Mulrow CD, et al. The PRISMA 2020 statement: an updated guideline for reporting systematic reviews. BMJ 2021;372:n71. doi: 10.1136/bmj.n71. This work is licensed under CC BY 4.0. To view a copy of this license, visit https://creativecommons.org/licenses/by/4.0/
